# Supplementary material for: Patterns of Intron Gain and Loss in Fungi
Source: PLoS Biol. 2004 Nov 30;2(12):e422. doi: 10.1371/journal.pbio.0020422 (PMC532390; doi:10.1371/journal.pbio.0020422)
Supplement: Table S1 — Also available at http://genes.mit.edu/NielsenEtAl/. (4.3 MB ZIP). [file pbio.0020422.st001.zip › NielsenEtAl/html/671.html]

AN5010.1.NCU05894.1.MG03271.1.FG06070.1


```
 CLUSTAL W (1.82) Multiple Sequence Alignments - Introns Inserted


Sequence 1: MG03271.1	411 aa
Sequence 2: FG06070.1	395 aa
Sequence 3: NCU05894.1	415 aa
Sequence 4: AN5010.1	371 aa
Alignment Length: 425 aa
Number Identitical Residues: 83 aa
Alignment Score (without introns) 5931


MG03271.1 	MAGVADKARFFLEQAVPQLREFEDKEIFTR0DEIRNLVRKRTDFEHRILAPGSKAPDFLA
NCU05894.1	MGSVAEKARFYLERAAPQLREFEEKEIFNK0DEIRSLVTKRSDFEHLVLSPGTKPSDFLS
FG06070.1 	MAGVAEKARFYLERAVPQLREWEDKEIFSK0DEIRTIVQKRNDYEHRVLSPGNRPSEWSS
AN5010.1  	MSATADKARFYLEQSVPELREYERKKIFSK0DEITSIIKKRSDFEHKINARGAQPADFVR
          	*...*:****:**::.*:***:* *:**.: *** .:: **.*:** : : * :..::  

MG03271.1 	~YADWERGLETLRARRCARLGIRQLTHQHAGVGRLGQIHERGVNRDPGSVHMWRSYLEHL
NCU05894.1	~YVQWERSMDRLRAKRCKRLKIRGSNPRPS-EARVFGIFERAVMKHPGCIPLWIAYLDFA
FG06070.1 	~YAQWEQSLESLRTKRCKRLKIRHLNSAHAGQGRTLAIYERGVNRHPGSSALWREYLSYI
AN5010.1  	2------------------------RQDSVGNVFRVFVLDRATRKFHGDIGLWVQYIEYA
          	                              .      : :*.. :  *   :*  *:.. 

MG03271.1 	SQQGAAKRWRRVATRALRLHPQDAELWTLVGRRSAA-NGDMDGARAYFLRGCRFCTKSVF
NCU05894.1	AEIKATKRWRRVITKAIRMHPRSAVLWTMAGRNAAV-HGDMERARAYFLRGCRFCTGEVS
FG06070.1 	SSVKASKRWRKTMTNALRMMPTDPELWAMAGRRSAK-NGDMAAARGFFMRGCRFCTTNEQ
AN5010.1  	RRQKAFKKLSTILMDALRLHPTKADLWIYAAHYSVEDHADMSQARTYMQRGLRFCKTSRK
          	    * *:       *:*: * .. **  ..: :. .:.**  ** :: ** ***. .  

MG03271.1 	IWVEYARVEMEWLAKVESRGKGK------KALAALRKEQ-SADDSDQIKLKDDSDESDDD
NCU05894.1	LWVEYARCEMEWLARIEAKKSGKGVRRGVNPIEAIKATEGTEIEGDHIALDDSDEDDEVD
FG06070.1 	LWVEYARSEMEWLEKVDKRKAEA------KPGQDVLRPD-REEEGDEMRLIDSDDEEEED
AN5010.1  	LWIQYAKLELIYITKLVARQRILG------------------LDEKPEGPKQQVSEDDLN
          	:*::**: *: :: ::  :    .                   : .     :. .:.: :

MG03271.1 	DDGNMIPDREMAAAKALERQQAPKLVDEEAVEKLEKSPALGGAIPRAIFDIATKQKFFEA
NCU05894.1	SDELILPDPDAGEKKKKPVFD------AEATRKLEQSPALSGAIPMAIFDIAKKQPFYAA
FG06070.1 	D----LPEPSNTQAKVIDKQ---------SAQQLKSNPAMDGALPMAIFDISKKQSFFGA
AN5010.1  	ADMIMMPQITEEDINPKARDDT---IDEAALKTLNSTPALTGAIPLAIFDSAIKTFDYND
          	 .   :*:      :     .:    .  : . *:..**: **:* **** : *   :  

MG03271.1 	TSAEAFFNMFSRFIFTVEPCADLIQHVLDTMMKSFERHPATWDCYIRQPLLPVQDFTTAD
NCU05894.1	AACEAFFDMFATFG-HVSCQSKITQHVLSAMEESFPKSACTASCFVRQPLIGVD-INTPE
FG06070.1 	NTAERFFNLFSTFT-KVPAQPRISQHVLAVLDQEYPNHPATCNVHIRQPIMGVN-PQTAE
AN5010.1  	CFAHEFFDMVFEFE-DVPCLQKILSHILDVMQESKPNSPHTLICYIKYPTAGVR-VTSAE
          	  .. **::.  *   *     : .*:* .: :.  . . *   .:: *   *    :.:

MG03271.1 	FPRALRESLGRLTK-GLEATDRKSELAGKTLSWVVSLLSKQGLDQSIETVLEMTKKKMES
NCU05894.1	FPKALRESLARLRKAYAEQTEDKKALAGKIVDWASKILEIVSLDPAIRTVLEATKKGLEA
FG06070.1 	FPKNLREVLVRLNK-YLETTTDPAELKKKTVAWIDGYLALDTLDEGIRAVLEHTKKKMEA
AN5010.1  	FPRALGAALSRLQE-----HRQKPQVAKEVIDWLRPVEKTKDLDPSIQKVIAATIHKAEL
          	**: *   * ** :           :  : : *         ** .*. *:  * :  * 

MG03271.1 	LL-----
NCU05894.1	ELGQEKA
FG06070.1 	I------
AN5010.1  	VLQEA--
          	   .
```
